# Targeting Cell-Specific Molecular Mechanisms of Innate Immunity in Atherosclerosis

**DOI:** 10.3389/fphys.2022.802990

**Published:** 2022-04-01

**Authors:** M. Sauter, H. F. Langer

**Affiliations:** ^1^ Cardioimmunology Group, Medical Clinic II, University Heart Center Lübeck, Lübeck, Germany; ^2^ Department of Cardiology, University Heart Center Luebeck, University Hospital, Luebeck, Germany; ^3^ DZHK (German Centre for Cardiovascular Research), Partner Site Hamburg/Lübeck/Kiel, Lübeck, Germany

**Keywords:** atherosclerosis, inflammation, immune system, dendritic cell, platelets, thrombosis

## Abstract

Mechanisms of innate immunity contribute to inflammation, one of the major underlying causes of atherogenesis and progression of atherosclerotic vessel disease. How immune cells exactly contribute to atherosclerosis and interact with molecules of cholesterol homeostasis is still a matter of intense research. Recent evidence has proposed a potential role of previously underappreciated cell types in this chronic disease including platelets and dendritic cells (DCs). The pathophysiology of atherosclerosis is studied in models with dysfunctional lipid homeostasis and several druggable molecular targets are derived from these models. Specific therapeutic approaches focussing on these immune mechanisms, however, have not been successfully introduced into everyday clinical practice, yet. This review highlights molecular insights into immune processes related to atherosclerosis and potential future translational approaches targeting these molecular mechanisms.

## Introduction

**FIGURE 1:**
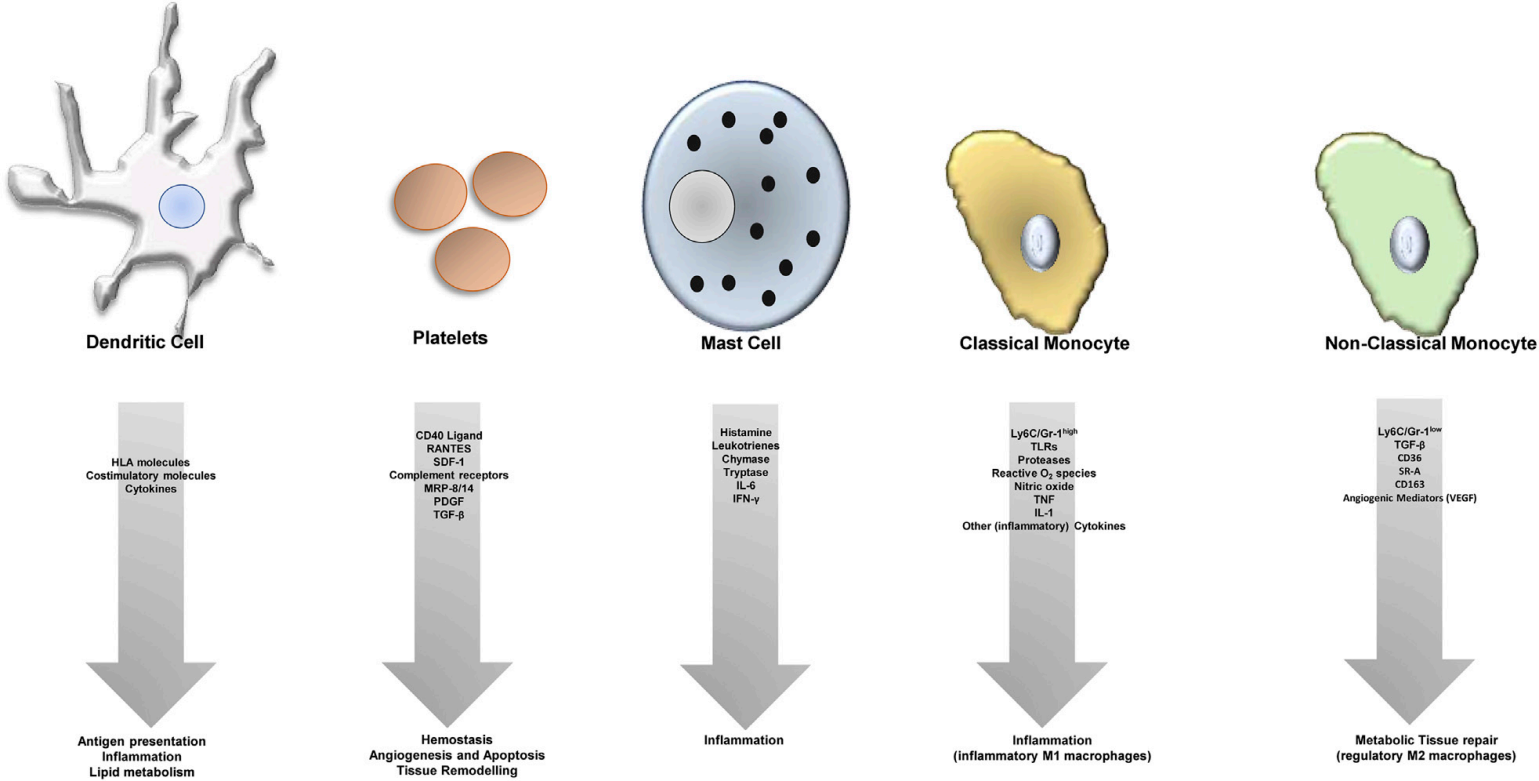
Different cell types contribute to atheroprogression through various (dysregulated) processes.

Atherosclerosis is an inflammatory disease, features a dysbalance in metabolism and together with its sequelay still has the largest contribution to deaths in developed countries—accounting for an estimated 17.9 million deaths every year (Collaborators, 2018; WHO CVD Risk Chart Working Group, 2019). The process of atherosclerotic plaque growth is initiated when cholesterol-containing low-density lipoproteins accumulate in the intima and activate the endothelium and subendothelial cells and structures (Libby et al., 2019). Subsequently, chemokines, endothelial and leukocyte adhesion molecules trigger recruitment of “classical” inflammatory cells related to atherosclerosis—monocytes and T cells (Gencer et al., 2021). Transformation of monocytes into macrophages is paralleled by internalization of lipoproteins, and foam cells develop (Bobryshev, 2006). Further release of cytokines, proteases, and vasoactive molecules from macrophages and foam cells is triggered by inflammatory signaling, lesional T-cells recognize local antigens and a T helper-1 response is generated with secretion of pro-inflammatory cytokines, which contributes to local inflammation and growth of the plaque (Frostegård et al., 1999; Ait-Oufella et al., 2011). This process requires co-stimulation for example, by the CD40-CD40L axis. At later stages, the plaque becomes vulnerable featuring local proteolysis, plaque rupture and finally thrombus formation with serious and potentially fatal events caused by ischemia and tissue infarction. Besides this classical picture, recently other cells have been centrally implicated in the process of atherogenesis (Figure 1) such as the classical antigen presenting cells of our body dendritic cells (DCs) or platelets, which are cells of the coagulation system.

Immunological memory was assumed to be an exclusive feature of the adaptive immune response for decades (Netea et al., 2020). But it was recently demonstrated that the innate immune system is able to generate non-specific memory responses, too—a phenomenon referred to as “trained immunity”. Innate immune cells were found to trigger a robust response to subsequent inflammatory challenges after initial activation by certain stimuli, for example, fungal-derived agents or vaccines (Penkov et al., 2019; Mitroulis I. et al., 2021). Surprisingly, macrophages can persistently switch their phenotype to be pro-inflammatory (M1-like) after a short stimulation with pathogens. These trained, pro-inflammatory macrophages show an pro-atherogenic phenotype and were detected in patients with established atherosclerosis as well as in humans after experimental infection for up to 3 months (Leentjens et al., 2018).

In this review, we focus on the influence of cell-cell interactions on atherogenesis and discuss the roles of mainly DCs and platelets in the context of the disease.

## Previously Underappreciated Immune Cell Types Contributing to Atherogenesis

Cells of both innate and adaptive immunity are known key factors for the initiation as well as the progression of atherosclerotic disease (Libby, 2002; Wolf and Ley, 2019a). One of the most investigated cell types involved in atherosclerosis are monocytes/macrophages, which can be classified into different phenotypic subsets (M1 and M2 Macrophages) that play oppossing roles in the progression of the disease (Murray and Wynn, 2011; Murray et al., 2014) by acting pro- (M1 macrophages) or anti-inflammatory (M2 Macrophages). During the last few years, it has been shown that vascular smooth muscle cells are able to differentiate into macrophage-like cells within atherosclerotic plaques (Rong et al., 2003; Feil et al., 2014; Shankman et al., 2015; Vengrenyuk et al., 2015). Macrophages develop to foam cells by lipid accumulation during the development of an atherosclerotic plaque (Randolph, 2014). These foam cells are thought to constitute the core of the atherosclerotic plaque and contribute to plaque destabilization. However, in recent years also other cells of innate immunity have entered the stage of research and gained increased attention as potential important contributors to the disease, like T-cells and dendritic cells (DCs).

T cells represent a group of cells of different phenotypes, which also play different roles in the disease process of atherosclerosis. For example, T helper1 cells (Th1) have a pro-atherosclerotic effect, while regulatory T cells (Tregs) play a more atheroprotective role. Tregs, however, can also “turn bad” and become pro-atherogenic (Wolf and Ley, 2019b; Saigusa et al., 2020).

DCs have been identified some years back as the classical antigen presenting cells of our body (Banchereau and Steinman, 1998). Interestingly, DCs were detected by several groups in high abundance within the vascular wall of humans and mice (Bobryshev and Lord, 1995; Choi et al., 2009). The frequency of their location within the vessel wall changes depending on the shear stress the area is exposed to ( Lord and Bobryshev, 1999; Yilmaz et al., 2004; Jongstra-Bilen et al., 2006 ). In the atherosclerotic ApoE knockout mouse model, accumulation of cholesterol in the cell membrane of DCs resulted in enhanced MHC-II-dependent antigen presentation and CD4^+^ T-cell activation (Bonacina et al., 2018). Whether DCs are protective or contribute to the extension of atherosclerotic plaques has been addressed in several studies, has surprisingly produced conflicting results and is, thus, still a matter of debate. A study by Gautier et al. using transgenic animals with BcL-2 expression under a CD11c promoter, which causes *in vivo* expansion of DCs, showed reduction of atherosclerosis, which suggests that DCs are protective (Gautier et al., 2009). Another study, however, which depleted DCs *in vivo* using the CD11c-diphteria toxin (DT) receptor model, has produced results with reduced atherosclerosis as well (Paulson et al., 2010). An explanation may be that repeated injections of DT is lethal for the animals (Jung et al., 2002; Probst et al., 2005).

Research on DCs in atherosclerosis carried out over the last decade reveals pro-as well as anti-atherogenic roles for DC subsets at all stages of atherogenesis explained by their numerous functions including lipid uptake, antigen presentation, efferocytosis, and inflammation resolution (Subramanian and Tabas, 2014; Zhao et al., 2021; Langer, 2022). Meanwhile, it is well established, that tolerogenic DCs, characterized by expression of CD103, act atheroprotective *via* induction of regulatory T cells (Hermansson et al., 2011). It has moreover been shown recently, that CD11c^+^ cells derived from murine bone marrow are able to efflux cholesterol and secrete ApoE which mediates an atheroprotective effect of these cells (Sauter et al., 2021).

When talking about DCs in atherosclerosis, it has to be considered that DCs represent a large group of cells whose precise definition is difficult because there is no specific, unique surface marker. CD11c, which is used predominantly as a “DC marker”, can also be expressed by macrophage subsets. Therefore, it is necessary to identify DCs by using additionally different cell-surface markers in parallel. DCs can be considered as CD11c^high^, MHC-II^high^ expressing cells that can be CD11b^±^, F4/80^±^, CD103^±^, DEC-205^±^, Clec9a^±^, and Mertklo/^−^. Other markers, namely Flt3, c-Kit, CD272, and CD26, which are expressed by classical DCs have been added in defining DC sub-populations, not only by classical flow-cytometry but also by “histo-cytometry”, in the last years (Gerner et al., 2012; Miller et al., 2012; Subramanian and Tabas, 2014).

Platelets are the most important cells of our body for wound sealing and initiation of the healing process after injury. Studies during the last two decades have moved platelets also more into the centre of inflammatory reactions (Koupenova et al., 2018). Some authors even suggest platelets to be part of the innate immune system (Croce and Libby, 2007). Interestingly, recent studies demonstrated the presence of platelets within the tissue after injury and their contribution to tissue remodeling processes such as apoptosis (Schleicher et al., 2015). In inflammation, platelets present receptors and release factors, which are important for the aggravation of the inflammatory response (Jenne et al., 2013). For development of atherosclerosis, the pontential significance of platelets has increased as studies some years ago demonstrated that platelet adhesion receptors or paracrine effectors can contribute significantly to plaque initiation and atherosclerotic inflammation (Massberg et al., 2002; Huo et al., 2003; Gawaz et al., 2005). Furthermore, at later stages of atherosclerosis, platelets are obviously crucial cells for the development of vessel occluding thrombi for example, in stroke or myocardial infarction patients, and targeting platelet associated mechanisms, which promote thrombus formation, is an approved approach to treat these patients (Nording et al., 2020).

Another immune cell type that is getting attention regarding its contribution to atheroprogression in the last years are neutrophil granulocytes (Döring et al., 2020). It has been shown before, that blood neutrophil counts in hypercholesterolaemic mice correlate with atherosclerotic lesion size (Drechsler et al., 2010). Neutrophils are able to form Neutrophil extracellular traps (NETs)—released decondensed chromatin that is decorated with granular proteins, forming a network of extracellular fibers (Brinkmann et al., 2004). NETs create a physical barrier that prevents the spread of pathogens and facilitates killing microbes by high concentrations of antimicrobial proteins and phagocytosis by other phagocytes (Brinkmann et al., 2004). NETs also promote thrombus formation (Laridan et al., 2019) and have been found in atherosclerotic plaques (Megens et al., 2012). The crosstalk of neutrophils with DCs can be realized *via* interactions between Mac-1 and DC-SIGN (van Gisbergen et al., 2005). Additionally, NETs and circulating immune complexes containing self-DNA and antimicrobial peptides were shown to trigger pDC activation *via* TLR9 (Lövgren et al., 2004; Means et al., 2005; Garcia-Romo et al., 2011; Lande et al., 2011). It was also reported that NETs in atherosclerotic lesions may stimulate a pDC-driven pathway of autoimmune activation and the generation of anti–double-stranded-DNA antibodies which may critically aggravate atherosclerosis lesion formation (Döring et al., 2012).

An interaction between neutrophils and platelets in atherosclerosis has been established. Activated platelets present HMGB1 to neutrophils, which causes them to perform autophagy and NET generation. This interaction may contribute to thromboinflammatory lesions (Maugeri et al., 2014). NETs are supposed to be involved at an early stage during the formation of coronary thrombus and lytic changes (Qi et al., 2017). A correlation between NET burden and DNase activity in ST-elevation acute coronary syndrome has been shown to be predictors of ST-segment resolution and infarct size (Mangold et al., 2015).

The role of dendritic cells (DCs) in atherosclerosis is controversial. DCs can present antigens to T cells, and may also shape plaque development by taking part in inflammatory processes as well as by influencing lipid metabolism. Platelets can interact directly with the endothelium and secrete different pro-atherogenic factors, which leads to dysfunction of the endothelium and recruitment of leukocytes. They furthermore play a significant role in immunity, express pro-inflammatory receptors (e.g., complement receptors) on their surface and contribute to atherosclerosis not only in their thrombus-forming function, but also *via* tissue remodelling, apoptosis and angiogenesis. Furthremore, mast cells take part in the plaque formation process and plaque growth by many different factors as indicated. Monocytes are the “progenitors” of macrophages. Macrophages turn into foam cells within plaques, the most prominent cell type within atheroclerotic lesions. Macrophages can be classified into a pro-inflammatory (regulatory, M2, Ly6C low expressing in mice), and anti-inflammatory cell (M1, Ly6C high expressing in mice), and express different cytokines or factors/mediators contributing to either inflammation (M1 macrophages) or tissue repair (M2 macrophages).

## Implication of Dendritic Cells in Atherogenesis

DCs are cellular components of the body`’s immune response connecting the innate and the adaptive immune system. First, immature DCs sample antigens from their surrounding environment, which is in case of vessels the blood stream or the vascular wall. Interestingly, in mice regions of arterial curvature and branch points exposed to disturbed blood flow show a relative abundance of DCs (Lord and Bobryshev, 1999), implying that they are present in vascular regions being predisposed for atherosclerosis. Similarly, at later stages of atherosclerotic lesion development in patients, high numbers of mature DCs can be detected (Yilmaz et al., 2004). If one looks at patients with stable or unstable angina pectoris or myocardial infarction, the number of DCs in the atherosclerotic plaque increases, and their prevalence was found to correlate with a concomitant decrease in the number of circulating DCs and DC precursors (Yilmaz et al., 2006b). It has to be mentioned, however, that the clear identification of DCs is complex, particularly as they have overlapping phenotypes and share surface receptors with other immune cells (Schraml and Reis e Sousa, 2015). Therefore, further studies are needed using additional markers, which have been meanwhile identified. Compared to DCs in healthy patients, those found in patients with unstable angina produce higher levels of the proinflammatory mediators TNF-alpha and IL-1beta (Ranjit et al., 2004), and hallmarks of cardiovascular inflammation including CRP and IL- 6 correlate inversely with the number of circulating DCs (Kretzschmar et al., 2012). This could implicate a direct asscociation of the inflammatory response mediated by DCs with these acute phase proteins or vice versa. When DCs are activated by foreign antigens, for example, in the skin, they migrate to regional lymph nodes and present the ingested and processed antigens *via* the major histocompatibility complex (MHC) on their surface together with supporting signalling receptors such as CD80, CD86, and CD40 (Fujii et al., 2004). This process then causes activation of helper T-cells and killer T-cells targeted against the presented antigen (Ni and O'Neill, 1997). For the vascular wall, these processes are not as well characterized and the role of (auto-)antigens for atherosclerosis is albeit very likely, not profoundly proven nor fully understood. There are, however, interesting new concepts and very promising approaches using for example, auto-antigens for “vaccination” against atherosclerosis such as MDA-LDL, oxLDL or native LDL (George et al., 1998; Asgary et al., 2007). This and the potential of autoantigens such as oxidized LDL or heat shock proteins to induce some kind of autoimmunity during the process of atheroprogression underlines the involvement of DCs in atherogenesis (Kurien and Scofield, 2008; Rahman et al., 2017). Vaccines for infectious diseases and cancer are designed to boost the pro-inflammatory and lytic T cell response. A vaccine for atherosclerosis treatment in contrast has to induce immunological tolerance and/or functional neutralization to support the inflammatory response. To achieve this, there are two main strategies: inducing B cell-dependent production of neutralizing antibodies (e.g., anti-PCSK9 or anti-oxLDL antibodies) or inducing a durable Treg or Th1 response. At this point, it has to be mentioned that the idea of a vaccination against atherosclerosis still leaves us with important questions (e.g., vaccine formulation, route of delivery, schedule and durability of vaccination, proper patient selection for testing, and monitoring of efficacy endpoints or safety issues), which will have to be answered in extensive preclinical and clinical studies (Chyu and Shah, 2018; Roy et al., 2020).

## How Metabolic Dysregulation Affects the Function of Innate Immune Cells Contributing to Atherosclerosis

The immune and metabolic response systems are linked to each other. They have evolved from common ancestral structures as shown by many evolutionary references (Leclerc and Reichhart, 2004). Obesity in humans leads to a dysregulation of the metabolic system resulting in a condition known as the “metabolic syndrome”. The metabolic syndrome causes a variety of pro-atherosclerotic effects on the arterial wall. Production of small dense LDL particles and decreased HDL levels increase vascular infiltration by lipids and the production of oxidiced LDL (ox-LDL). Ox-LDL delivers a danger signal to macrophages triggering development of foam cells, which in turn produce cytokines and growth factors for the growing atherosclerotic plaque. In addition, elevated blood levels of cytokines and of adipokines contribute to aggravation of the inflammatory reaction (Mathieu et al., 2006). In obese patients, a shift in macrophages towards a M1-like phenotype can be observed, which is caused by increased levels of free fatty acids (FFAs), cholesterol, LPS, and hypoxia (Kim et al., 2010), thereby further contributing to adipose tissue (AT) inflammation and inhibition of insulin signaling, whereas in lean individuums the anti-inflammatory M2-like macrophage phenotype prevails (Lumeng et al., 2008; Sun et al., 2021a). From a clinical prespective, it is undoubted and well established for decades that diabetes and atherosclerosis are linked, as the cardiovascular disease (CVD) risk is increased two-to-four-fold in diabetic persons compared to non-diabetic persons (Kannel and McGee, 1979). Importance of a metabolism—immune crosstalk involving DCs is documented by the fact that application of statins leads to a lower number of dendritic cells and less mature DCs in atherosclerotic plaques, representing one of the potential pleotropic anti-atherosclerotic effects of these clinically established substances (Yilmaz et al., 2006a). Interesintgly, statins not only reduce the risk of CV events by having an impact on cytokines such as CRP or IL-6 (Arnaud et al., 2005), but directly influence DC invasion (Kofler et al., 2008).

Dyslipidemia is one, but not the only epigenetic regulator that influences the action of antigen-presenting cells in the course of atherogenesis. Additionally, there exists a “response-to-injury hypothesis of atherosclerosis” (Glagov et al., 1987), which initially proposed that endothelial denudation is the first step in atherosclerosis and where activation and damage of the endothelial monolayer caused, for example ,by previous severe conditions including trauma, are supposed to effect a pool of endothelial progenitor cells and mono-nuclear progenitors in tissue reparation, triggering the development of the lesions (Du et al., 2012). It has been furthermore shown that metabolic changes can influence the phenotype and function of DCs (Sun et al., 2021b). Studies with patients suffering from diabetes mellitus type 1 and type 2 revealed that previous episodes of hyperglycemia can have a long-standing impact on the subsequent development of cardiovascular diseases—a phenomenon known as “metabolic memory” (Control et al., 2003; Holman et al., 2008). It has been postulated that epigenetic mechanisms may participate in conferring this metabolic memory (El-Osta et al., 2008). *In vitro* studies with aortic endothelial cells showed that transient incubation in high glucose followed by subsequent return of these cells to a normoglycemic environment was associated with increased gene expression of the p65 subunit of NF-κB, NF-κB activation, and expression of NF-κB–dependent proteins, including MCP-1 and VCAM-1(El-Osta et al., 2008).

## DCs, Tregs and Atherosclerosis

DCs contribute to vascular inflammation by presentation of costimulatory molecules and subsequent signalling in immune cells, their activity may however be also controlled by T regulatory cells (TRegs (Lutgens et al., 2010). The main functions of Treg cells, which originate from CD4^+^ T cells, are the control of autoimmunity and the maintenance of self-tolerance. By interacting with DCs or their inhibition, Tregs are able to suppress the activation of effector T cells. This regulates priming and execution of T-effector responses (Ait-Oufella et al., 2014). Various cDC subtypes modulate Treg cell homeostasis in different ways (Roy et al., 2021), and Tregs are also involved in the activation, maturation, and function of DCs(Steinman and Banchereau, 2007; Chistiakov et al., 2013). T cell activation by DCs is dependent on toll-like receptor (TLR)-mediated maturation of DCs (Roy et al., 2021). In one study by Subramanian et al., bone marrow was transplanted from mice with CD11c^+^ DCs deficient in the TLR adaptor MYD88 into LDL receptor knockout mice, which led to decreased recruitment of both effector T cells and Treg cells to atherosclerotic lesions. The transplanted mice showed significantly larger atherosclerotic lesion sizes, propably caused by increased production of the monocyte-recruiting chemokine CCL2 due to the loss of Treg cells (Subramanian et al., 2013). Other studies have shown that the chemokines CCL17 and CCL22, secreted by tolerogenic CDs, can lead Tregs to atherosclerotic lesions. There, Tregs could suppress immunomodulatory properties of proinflammatory DCs and prevent differentiation of immature DCs to inflammatory subsets of mature DCs (Iellem et al., 2001; Weber et al., 2011; Chistiakov et al., 2014).

## Platelets—Atherosclerosis

Platelet mediated thromboinflammation is an important cornerstone of atherosclerosis. Recently, an expert Consensus Document from the Third Maastricht Consensus Conference on Thrombosis was published, which focused both on the cellular and soluble parts of coagulation and their impact on inflammation in cardiovascular disease (d'Alessandro et al., 2020). Platelets are connected to vascular inflammation by adhesion receptors. They have two main classes of agonist receptors, G protein-coupled receptors (GPCRs) and immunoreceptor tyrosine-based activation motif (ITAM)-containing receptors, which sense changes in the environment and thereby initiate intracellular signaling required for integrin activation, which in turn is the major mechanism for attachment during thrombus formation (Bergmeier and Stefanini, 2018). Particularly, GPCRs form a “G protein highway to integrin activation”, which is crucial for classical hemostasis, as evidenced by severe bleeding phenotypes after genetic disruption or pharmacological intervention with individual components of this pathway. In contrast, ITAM receptors play a less important role during classical hemostasis but are considered to be more important for thromboinflammation, when platelet aggregation under flow does not happen (d'Alessandro et al., 2020).

The platelet collagen receptor GPVI has attracted attention, as clinical trials targeting this receptor were recently initiated or completed (Voors-Pette et al., 2019). Inhibition of this receptor using Revacept®, a lesion-directed antithrombotic drug binding to exposed collagen of the atherosclerotic plaque, was analyzed in a phase 2 randomized clinical trial with patients undergoing elective percutaneous coronary intervention (PCI) for stable ischemic heart disease (Mayer et al., 2021). As expected, bleeding was not increased and, the authors analyzed a short-term endpoint of a composite of death or myocardial injury, whereby Revacept® had no significant effect.

Another important mechanism, how platelets contribute to ahterosclerosis is their ability to form platelet–neutrophil hetero-aggregates, which are mediated through adhesion molecules such as P-selectin and P-selectin glycoprotein ligand 1 as well as glycoprotein Ib and macrophage-1 antigen (Evangelista et al., 1999; Zarbock et al., 2007; Sreeramkumar et al., 2014b), which then activates intracellular signaling. As a consequence, soluble mediators are released and also direct signaling between platelets and neutrophils is initiated, which leads to their reciprocal activation and neutrophil release of extracellular traps (NETs). These Nets are scaffolds of condensed chromatin, which are exremely prothrombotic and, thus, mediate atherothrombosis (Döring et al., 2017).

Complement—a central part of innate immunity—plays an important role in the progression of atherosclerosis (Haskard et al., 2008). Platelets have been shown to express receptors for complement factors on their surface. For instance, it was demonstrated that the expression of anaphylatoxin-receptors (C3aR and C5aR) and activation markers (i.e., P-selectin) on platelets correlate in CVD patients (Patzelt et al., 2015). It has been shown furthermore, that the complement anaphylatoxin C5a inversely correlates with platelet bound oxLDL (Nording et al., 2016). Complement receptor expression on platelets has recently been shown to play important roles in thrombosis (Sauter et al., 2018) and neovascularization (Nording et al., 2021). Furthermore, C3 can activate platelets independent of formation of the terminal complement complex, which provides evidence for the contribution of complement-dependent membrane perturbations *in vivo* to prothrombotic TF activation on myeloid cells (Subramaniam et al., 2017). Together, complement factors may present a promising target for therapeutic approaches in the prevention and treatment of atherosclerosis which will have to be addressed in future studies.

## Platelets, DCs—Atherosclerosis

**FIGURE 2:**
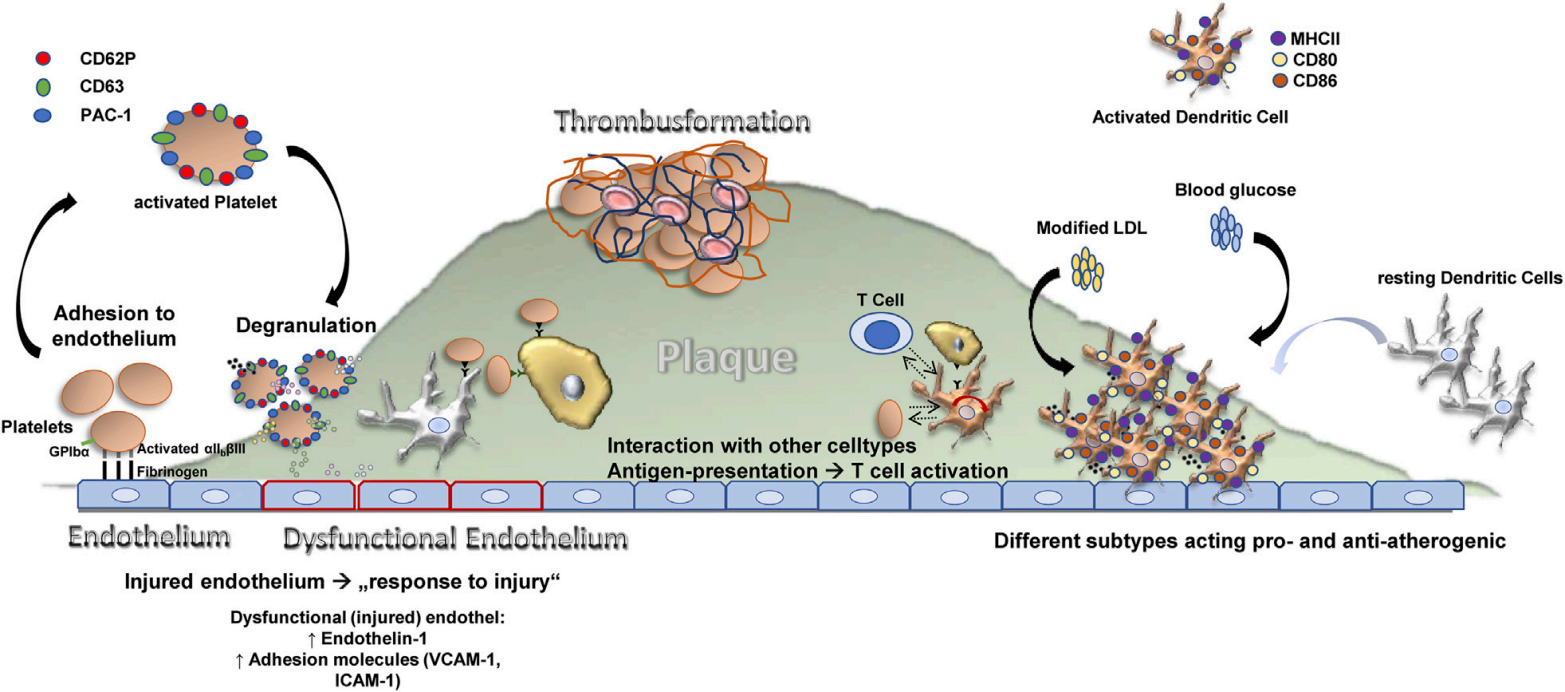
The contribution of DCs and platelets to atherosclerosis.

The interaction of immune cells and platelets has been suggested to be of importance in atherogenesis and atheroprogression (Figure 2) (Massberg et al., 2002; Nording et al., 2015). DCs are able to recruit platelets *via* CD11b/CD18 (Mac-1) and platelet JAM-C, which leads to DC activation and platelet phagocytosis—a process that may be important for the progression of atherosclerotic lesions (Langer et al., 2007). On the other hand, platelets are able to activate Mac-1 on leukocytes (Sreeramkumar et al., 2014a). Recently, we discovered a further functional crosstalk of platelets with DCs mediated by GPIbα, which causes activation of beta2 integrins on the immune cells, involving a PSGL1 dependent mechanism (unpublished data). Another study by Verschoor et al. showed, that platelets and CD8α^+^ DCs are also able to interact *via* GP1b and complement component C3 (Verschoor et al., 2011). Additionally, platelets induce the secretion of pro-inflammatory cytokines from leukoctyes by providing neutrophil and endothelial activators, and thereby drive the process of acute inflammation (Zarbock et al., 2007).

Platelets shape atherosclerosis by their adhesion to the endothelium, secretion of pro-atherogenic factors such as PF-4, SDF-1, RANTES, CD40L, IL-1β as well as by interaction with other celltypes and leukocyte recruitment which leads to inflammation and thrombus formation. Dendritic cells differentiate into activated DCs in an atherosclerotic environment and accumulate in the growing plaque. There, accumulating evidence suggests that they shape atherosclerosis *via* parakrine effectors, intracellular signalling and interaction with other celltypes. The net effect of DCs on atherosclerosis still is a matter of debate.

A receptor, which belongs to the tumor necrosis factor family of ligands and is interesting for the topic of this review, as it is expressed on both platelets and T cells is CD40 ligand (CD40L), which was described to be relevant for atheroprogression in a cell specific manner. CD40L is a costimulatory molecule and together with CD40 its corresponding receptor on DCs is responsible for T-cell priming during antigen presentation of infectious agents (Grewal and Flavell, 1996). Not only T cells, but also monocytes, macrophages and endothelial cells express CD40L indicatíng that this protein has a broader function *in vivo*. The fact that CD40L is upregulated on the surface of activated platelets has attracted attention, as platelet derived CD40L can activate endothelium and therey contribute directly to the inflammatory response at the vessel wall for example, during the development of atherosclerosis (Lievens et al., 2010). For thrombus formation, CD40L was shown to stabilize in a beta3 integrin--dependent manner *in vivo* and to induce platelet spreading and aggregation (Yacoub et al., 2010). As T-cells and platelets are crucial already for early steps of atherosclerosis, the predominant cell-specific role of CD40L was recently addressed generating platelet- and T cell specific CD40L knockout mice. With this approach it could be demonstrated that platelet CD40L mainly is important for atherothrombosis, wheras T cell CD40L has a major function for the development of atherosclerotic plaques, while platelet CD40L did not affect this aspect (Lacy et al., 2021).

## Potential Therapeutic Applications

Lipid-lowering statin therapy is the standard therapy for primary and secondary prevention of cardiovascular diseases. But atherosclerosis is considered to be an inflammatory disease—therefore, anti-inflammatory approaches are promising targets in fighting atheroprogression.

One therapeutic aim is to reduce inflammation without affecting lipid levels, which was supposed to reduce the risk of cardiovascular disease: It has been shown that an anti-inflammatory therapy with canakinumab targeting the interleukin-1β innate immunity pathway could be promising, which led to a significantly lower rate of recurrent cardiovascular events than placebo, independent of lipid-level lowering (Ridker et al., 2017). Another milestone that supports the role of inflammation as a key mediator in the development of cardiovascular disease was a study with colchicine, where in patients with chronic coronary disease the risk of cardiovascular events was significantly lower with colchicine compared to those who received placebo (Nidorf et al., 2020). The discovery of trained immunity mentioned in the beginning of this review may be an attractive target for future therapeutic interventions or even vaccine strategies as it shows common mechanistic pathways with cardiometabolic disease, including epigenetic changes or macrophage activation in response to cytokines (Davis and Gallagher, 2019; Netea et al., 2020; Mitroulis i. et al., 2021). The activation of Tregs by tolerogenic DCs could also provide translational potential, because this interaction inhibits the inflammatory reaction within the lesions. To this end, focus should be put on the expansion of this DC subtype, as vaccination studies already showed promising effects (Hermansson et al., 2011, Nettersheim et al., 2020).

## Conclusion

The role of macrophages, which develop into foam cells contributing to plaque build-up is well established. Recent insights highlight the role of previously underappreciated cells in this context including platelets or dendritic cells as well as their interaction for the pathogenesis of atherosclerosis. Cellular, paracrine mechanisms and crosstalk with other important pathophysiological principles such as metabolic dysregulation have been uncovered and further underline the translational potential of and urgent need for characterizing new players and underlying mechanisms in atheroprogression.
